# Incorporating knowledge of disease-defining hub genes and regulatory network into a machine learning-based model for predicting treatment response in lupus nephritis after the first renal flare

**DOI:** 10.1186/s12967-023-03931-z

**Published:** 2023-02-03

**Authors:** Ding-Jie Lee, Ping-Huang Tsai, Chien-Chou Chen, Yang-Hong Dai

**Affiliations:** 1 Division of Nephrology, Department of Internal Medicine, Tri-Service General Hospital, National Defense Medical Center, Taipei, Taiwan; 2 Department of Internal Medicine, Tri-Service General Hospital Songshan Branch, National Defense Medical Center, Taipei, Taiwan; 3 Department of Radiation Oncology, Tri-Service General Hospital, National Defense Medical Center, Taipei, Taiwan

**Keywords:** Lupus nephritis, Renal flare, Transcriptomics, Machine learning, Prediction model

## Abstract

**Background:**

Identifying candidates responsive to treatment is important in lupus nephritis (LN) at the renal flare (RF) because an effective treatment can lower the risk of progression to end-stage kidney disease. However, machine learning (ML)-based models that address this issue are lacking.

**Methods:**

Transcriptomic profiles based on DNA microarray data were extracted from the GSE32591 and GSE112943 datasets. Comprehensive bioinformatics analyses were performed to identify disease-defining genes (DDGs). Peripheral blood samples (GSE81622, GSE99967, and GSE72326) were used to evaluate the effect of DDGs. Single-sample gene set enrichment analysis (ssGSEA) scores of the DDGs were calculated and correlated with specific immunology genes listed in the nCounter panel. GSE60681 and GSE69438 were used to examine the ability of the DDGs to discriminate LN from other renal diseases. K-means clustering was used to obtain the separate gene sets. The clustering results were extended to data derived using the nCounter technique. The least absolute shrinkage and selection operator (LASSO) algorithm was used to identify genes with high predictive value for treatment response after the first RF in each cluster. LASSO models with tenfold validation were built in GSE200306 and assessed by receiver operating characteristic (ROC) analysis with area under curve (AUC). The models were validated by using an independent dataset (GSE113342).

**Results:**

Forty-five hub genes specific to LN were identified. Eight optimal disease-defining clusters (DDCs) were identified in this study. Th1 and Th2 cell differentiation pathway was significantly enriched in DDC-6. *LCK* in DDC-6, whose expression positively correlated with various subsets of T cell infiltrations, was found to be differentially expressed between responders and non-responders and was ranked high in regulatory network analysis. Based on DDC-6, the prediction model had the best performance (AUC: 0.75; 95% confidence interval: 0.44–1 in the testing set) and high precision (0.83), recall (0.71), and F1 score (0.77) in the validation dataset.

**Conclusions:**

Our study demonstrates that incorporating knowledge of biological phenotypes into the ML model is feasible for evaluating treatment response after the first RF in LN. This knowledge-based incorporation improves the model's transparency and performance. In addition, *LCK* may serve as a biomarker for T-cell infiltration and a therapeutic target in LN.

**Supplementary Information:**

The online version contains supplementary material available at 10.1186/s12967-023-03931-z.

## Background

Lupus nephritis (LN) is a severe complication of systemic lupus erythematosus (SLE). LN is a form of glomerulonephritis and is typically classified into six distinct histological classes depending on the manifestation and severity of renal involvement [1]. Even though there have been accumulating knowledge and effective therapeutic options in recent decades, LN remains a clinical challenge [2–4]. With current treatments such as glucocorticoids, cyclophosphamide (CYC), or mycophenolate mofetil (MMF), less than 50% of patients achieve a complete clinical response after 1 year [5]. Even with clinical remission, 44.4% of patients show residual histological activity, and 27–66% develop renal flare (RF) [6, 7]. Once RF occurs, the risk of progressive kidney disease is dramatically increased, leading to poor outcomes and greater economic burden [8–10]. Thus, the prevention of RF with appropriate maintenance immunosuppressive therapy is vital and may decrease long-term morbidity and mortality [7]. However, 5–30% of patients develop end-stage kidney disease (ESKD) within 10 years [5, 11]. This discrepancy in therapeutic response among patients with LN indicates that the effect of conventional immunosuppressive drugs is not uniform, and the identification of candidates responsive to therapies is necessary for personalized medicine.

As histological changes are limited in LN, depending on the histological classification for therapeutic assessment is unreliable. In fact, the response to therapy might be potentially affected by intrarenal molecular mechanisms that drive disease through specific pathogenic pathways [12]. Currently, biomarkers for predicting treatment response in LN are accumulating but are mainly focused on serum and urinary analysis [13–15]. According to a recent systematic review, there was vast heterogeneity across studies, limiting their use in clinical settings [16]. Further, as the exploration of renal tissue is the gold standard for LN diagnosis, obtaining genomic information to identify the contributing disease pathways may provide the best value in predicting treatment response.

To the best of our knowledge, few studies have assessed large-scale transcriptomic profiles of LN under varying conditions. Mejia-Vilet et al. extracted RNA from kidney biopsies and found that intrarenal immune gene expression differed between LN at diagnosis and at flare [17]. Recently, Parikh et al. adopted serial renal biopsies and conducted extensive transcriptomic analyses to dissect the immune pathways responsible for determining drug responses after RF, providing insights into this clinical scenario [12]. In fact, their findings could be extended if the biological pathways responsible for drug response and genes involved in LN were connected. This approach of incorporating knowledge relevant to disease could help eliminate false-positive markers and enhance the signal-to-noise ratio in large-scale omics data [18, 19].

In this study, we aimed to construct prediction models for treatment response in patients with LN after the first RF. We attempted to identify disease-defining genes (DDGs) for LN and incorporated these genes into the feature selection process for model establishment.

## Methods

### Study samples

Transcriptomic data files were downloaded from the National Center for Biotechnology Information Gene Expression Omnibus (GEO; http://www.ncbi.nlm.nih.gov/geo) using the R/Bioconductor *GEOquery* package. DNA microarray data from GSE32591 and GSE112943 were used as discovery datasets to identify hub genes for LN. Data from GSE32591 (Affy_HGU133A_CDF_ENTREZG_10) were extracted from glomerular and tubulointerstitial compartments in 64 patients with LN and 29 healthy controls and normalized using the robust multi-array average (RMA) [20]. Data from GSE112943 (Illumina HumanHT-12 V4.0 expression beadchip) were based on kidney samples and contained 14 LN cases and seven controls [21]. The expression data were normalized using variance-stabilizing transformation and robust spline normalization. To confirm the disease-defining value of the hub genes, gene expression data derived from blood samples of patients with LN were used. GSE81622 (Illumina HumanHT-12 V4.0 expression beadchip), GSE99967 (Affy_HuGene-2_0-st), and GSE72326 (Illumina HumanHT-12 V4.0 expression beadchip) were included, and the data were normalized according to the methods described by the authors. Blood samples from 58 patients with LN and 63 healthy controls were evaluated. Further, GSE60861 (Agilent-026652 Whole Human Genome Microarray 4 × 44 K v2) and GSE69438 (Affy_HG-U133_Plus_2) consisted of gene expression data from chronic renal diseases in addition to LN [22–24]. Therefore, these two datasets were used to examine the impact of hub genes in the discrimination of LN from other kidney diseases.

For the construction of prediction models, data from a Series Matrix File (GSE200306) that evaluated the treatment response in patients with LN at the first RF was used [12]. This study included 58 patients with proliferative LN (class III or IV + / − V) and ten healthy subjects. Treatment for LN at RF consisted of induction therapy followed by maintenance therapy (a combination of prednisone and CYC or MMF). Response was assessed after the completion of the induction therapy, and responder was defined as the reduction of proteinuria greater than 0.5 g/d with improved serum creatinine. A second biopsy was performed within 1 year. Another study (Series Matrix File: GSE113342) conducted by Mejia-Vilet et al. compared the transcriptomic profile at RF and the expression at de novo LN [17]. Fourteen patients with LN with two tissue compartments (glomeruli and tubulointerstitia) were included, and 28 samples at RF were used for validation. Both studies adopted the same immunology gene panel (nCounter NanoString Human Immunology v2), which included over 500 general immunology genes, including major classes of cytokines and their receptors, enzymes with specific gene families, interferons and their receptors, the tumor necrosis factor-receptor superfamily, and the Killer-cell immunoglobulin-like receptor family genes. Additional 84 genes related to anti-fungal immune response are also included. This panel is therefore ideal for studying immune-related conditions and diseases. A key advantage of this NanoString technology is the ability to directly quantify molecules of interest in the absence of an amplification step, which prevents introduction of artificial bias [25]. Gene expression data were pre-processed and normalized using author-defined methods. The details of the datasets are provided in Additional file 1: Table S1 and Additional file 2: Figure S1.

### Identification of differentially expressed genes between LN and healthy control

Principal component analysis (PCA) was performed to examine the altered gene expression profile of LN in the discovery datasets. The first two components that covered most of the variance were used to generate the PCA plots. As two tissue components were identified in the GSE32591 dataset, the analyses were separated. The R/Bioconductor *ComplexHeatmap* package was used to create heat maps with hierarchical clustering. Differentially expressed genes (DEGs) were identified using a linear model derived from the R/Bioconductor *limma* package. A Benjamini-Hochberg (BH) adjusted P < 0.05 was considered significant. Venn diagram analysis (https://bioinformatics.psb.ugent.be/webtools/Venn/) was performed to identify common overlapping DEGs between the two tissue compartments in the GSE32591 and GSE112943 datasets. Over-representation analysis (ORA) was applied to identify significantly enriched Gene Ontology (GO) terms using Metascape (https://metascape.org/). DisGeNET (https://www.disgenet.org/) was used to assess the association between the genotypes and disease phenotypes. The adjusted P-value threshold was set at 0.05.

### Identification of DDGs for LN

A protein–protein interaction (PPI) network was constructed by applying the up-regulated and down-regulated DEGs in the Search Tool for the Retrieval of Interacting Genes (STRING). The network was reconstructed using Cytoscape software (version 3.8.2). The top sub-network modules were selected using plug-in molecular complex detection (MCODE). The criteria for determining DDGs for LN were an MCODE score > 3 and node number > 5. Top-ranked modules were chosen as the regulatory networks for the up-regulated and down-regulated genes. CytoHubba was used to identify the central elements of the biological networks [26]. The top 10 nodes from the algorithms, namely, the maximum neighborhood component (MNC), maximal clique centrality (MCC), edge percolated component (EPC), density of maximum neighborhood component (DMNC), and degree, were selected, and those with a degree less than ten were excluded. The blood samples in GSE81622, GSE99967, and GSE72326 were used to evaluate the impact of the DDGs. The P-value was calculated using Wilcoxon’s rank sum test and was log10-transformed.

### Permutation test

To confirm the significance of DDGs as gene signatures for LN, a permutation test was conducted. The same number of genes was randomly selected, and single- sample gene set enrichment analysis (ssGSEA) score was calculated for each sample. This process was repeated 1000 times to generate a background distribution of ssGSEA scores, which were then compared across LN samples and controls. Differences in the mean values of ssGSEA scores were obtained, and Wilcoxon’s rank sum test was used to derive the P-values. If there was a significant difference in the mean values and a low P-value against the background of our gene signature, it was considered significant.

### Identification of disease-defining clusters (DDCs)

The ssGSEA score was calculated to estimate the enrichment of hub genes in each sample. Spearman’s correlation coefficients (rho) were calculated between the hub gene ssGSEA score and the expression of each immunology panel gene. The coefficients of tissue and blood samples were pooled together, followed by K-means clustering to identify clusters in the immunology gene panel. The optimal number of clusters was determined using the elbow method. Genes in each cluster were further characterized by GO and Kyoto Encyclopedia of Genes and Genomes (KEGG) pathway enrichment analyses implemented in STRING [27]. DEGs in the immunology gene panel between responders and non-responders in GSE200306 were identified through a linear model using the *limma* package with the same criteria for significance mentioned above. To evaluate the influence of the clusters on the treatment response, the overlap of genes in a cluster with the DEGs for response was performed. Next, the least absolute shrinkage and selection operator (LASSO) was adopted to select genes with contributory effects to the response in a target cluster using the *glmnet* package in R. The selected genes were evaluated using CytoHubba and ranked according to the five network scores. The top regulatory genes were defined as high-rank genes with significantly different expression levels between responders and non-responders.

### Evaluation of immune cell infiltration

Because the top regulatory genes were extracted from the nCounter immunology panel, they may potentially affect immune cell infiltration. To estimate immune cell infiltration for each sample, immune cell type enrichment analysis was carried out in the discovery sets and blood samples. ssGSEA scores for immune cell-specific gene sets were calculated using the *ConsensusTME* package in R [28]. The immune signatures used in our study have been proposed by xCell, Bindea et al., and Danaher et al. [29–31]. Spearman’s coefficient was calculated between the expression of the top-ranked regulatory genes and the ssGSEA score, which estimates immune cell infiltration.

### Construction of prediction models

Patients with LN at the first RF were randomly divided into training and testing sets based on an 8:2 ratio. The original ratio of responders to non-responders was 2.76:1 in the GSE200306. Therefore, the synthetic minority oversampling technique (SMOTE) in the training set was performed to balance the minority group using the *DMwR* package. The LASSO regression algorithm was used to obtain genes with non-zero coefficients via tenfold cross-validation. Receiver operating characteristic (ROC) analysis with area under the curve (AUC) and 95% confidence interval (CI) was performed to evaluate the performance of the LASSO models using the *pROC* package in R in the training, testing, and entire sets. Patients with LN at RF in GSE113342 were used as a validation set to examine the predictive ability of the LASSO models, which was evaluated using the confusion matrix, precision, recall, and F1 score.

### Statistical analysis

All statistical analyses were conducted using the R software (version 4.1.2). Wilcoxon’s rank sum test was performed for continuous variables, and P < 0.05 was set as the threshold for statistical significance.

## Results

**Fig. 1:**
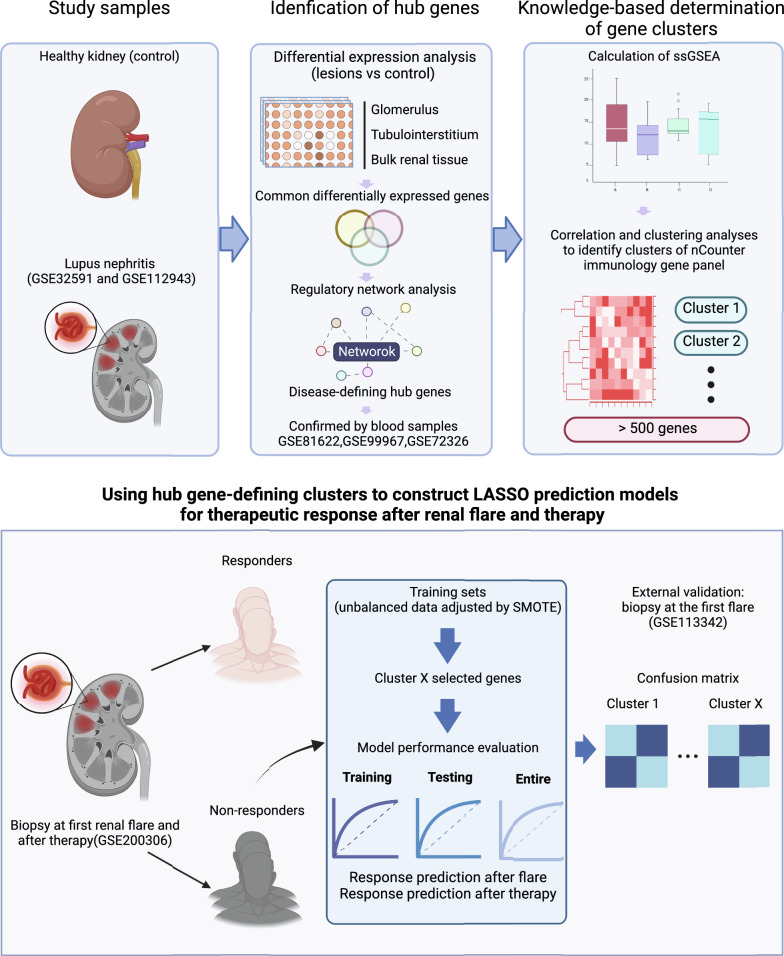
Study design and bioinformatic pipeline. This graph was created with BioRender.com (license number IB24MA3A0V)

A comprehensive analysis was conducted in this study. The overall design and flowchart of the study are presented in Fig. 1.

### Transcriptomic profiling in discovery datasets reveals alteration of gene expression in LN

**Fig. 2:**
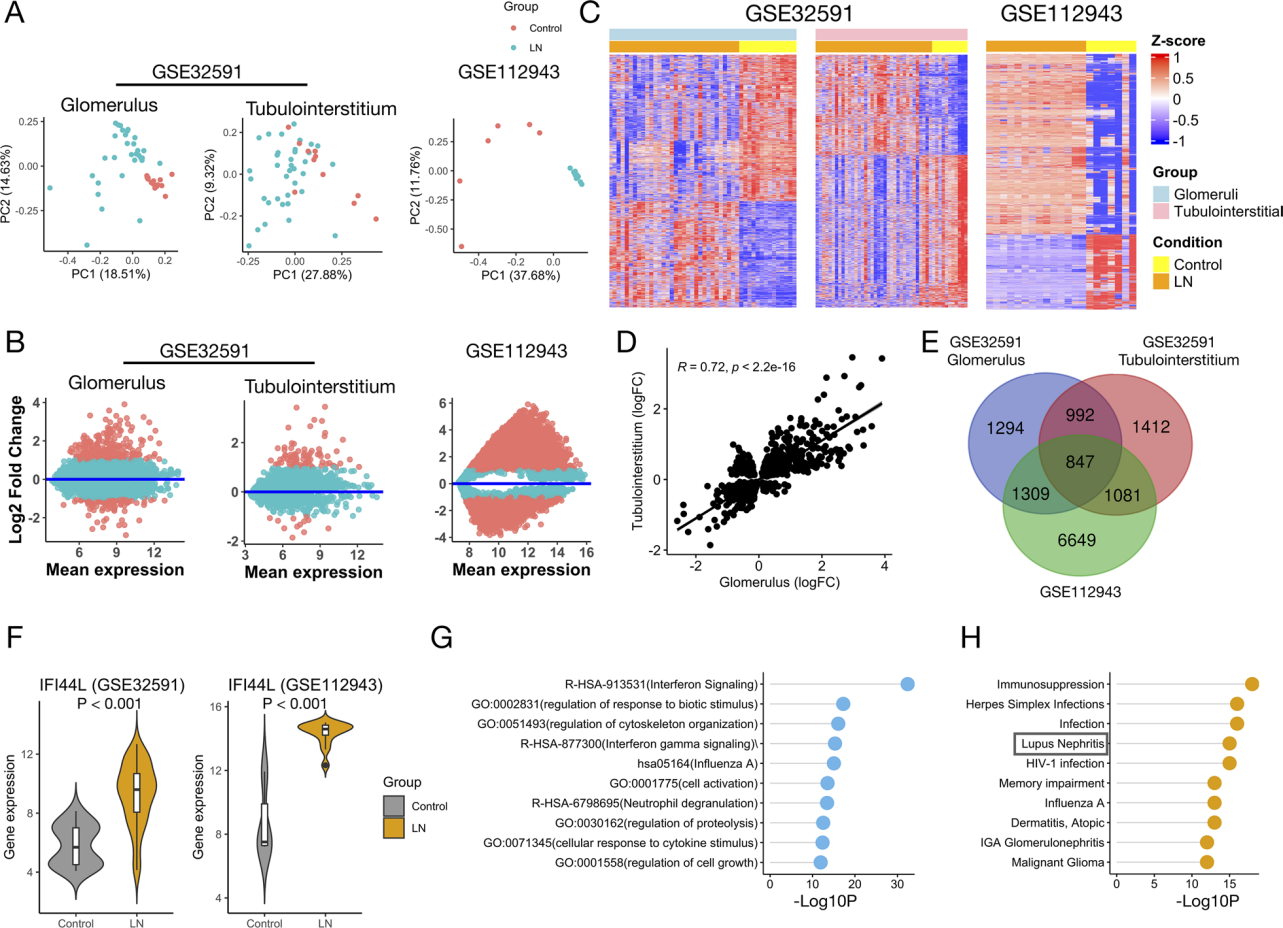
Characterization of transcriptomic profiles in LN. **A** PCA of genes between LN samples and healthy controls in GSE32591 and GSE112943. GSE32591 is divided according to tissue origins to evaluate the expression patterns in two tissue compartments. Aquamarine: LN samples. Red: healthy controls. **B** MA plots visualize the DEGs between LN and control samples. The data are transformed into M (logFC) and A (mean expression). Red dots indicate DEGs with |logFC ≥ 1|. Aquamarine dots indicate DEGs with |logFC < 1|. **C** Heatmaps of up- and down-regulated genes in LN. Clusters of genes are stratified using hierarchical clustering. Expression levels are z-transformed. Color bar indicates the transformed expression value. **D** Scatter plot of correlation between logFC of common DEGs in glomerular and tubulointerstitial compartments. Significance of correlation is performed by Spearman’s test. **E** Venn diagram of DEGs among two tissue compartments in GSE32591 and renal tissue in GSE112943. **F** Violin plots reveal differences in *IFI44L* expression between LN samples and controls in GSE32591 (left panel) and GSE112943 (right panel). Wilcoxon’s rank sum test P value is shown. **G** Dot plot reveals clustered enrichment ontology categories from ORA. –Log10-transformed multiple testing-adjusted P value is shown for each enriched term. **H** Dot plot reveals the significance of association with diseases via DisGeNET. Log10-transformed multiple testing-adjusted P value is shown for each enriched phenotype

To determine whether the gene expression levels could separate the study subjects into distinct groups, PCA was performed using GSE32591 and GSE112943. Striking clustering patterns were identified, whereby the normal glomerular samples in GSE32591 and the LN samples in GSE112943 showed distinct clustering (Fig. 2A). On the other hand, the subgroup of tubulointerstitial samples with LN clustered with healthy controls. To specifically examine the clustering patterns, a DEG analysis was conducted. To obtain all DEGs, no threshold for logFold Change (FC) was set, and Venn diagram analysis was performed to obtain common DEGs. A total of 4442, 4332, and 9886 DEGs were found in the glomeruli (GSE32591), tubulointerstitia (GSE32591), and renal tissues (GSE112943), respectively (Fig. 2B). The heatmap analysis supported the findings of PCA analysis, and part of the tubulointerstitial tissues with LN (n = 4) shared DEG expression patterns similar to those of the controls (Fig. 2C). In addition, one healthy control showed a DEG expression pattern similar to that of LN in GSE112943. To generate discovery datasets for comparison in an unbiased manner, the five samples were removed. Moreover, 1839 DEGs were shared between glomeruli (1839/4442, 41.4%) and tubulointerstitia (1839/4332, 42.5%), and the common DEGs were highly correlated (Spearman’s rho = 0.72, P < 2.2e-16) (Fig. 2D). This suggested that larger parts of the pathogenesis pathways were shared, and the information on genes relevant to LN could be derived from both tissue components. Venn diagram analysis revealed 847 common DEGs among the three subgroups of the discovery dataset (Fig. 2E). Among these, *IFI44L*, which was previously identified as one of six common DEGs (*IFI27*, *IFI44*, *IFI44L*, *IFI6*, *EPSTI1*, and *OAS1*) between SLE and normal samples, was the most common top DEG in GSE32591 and GSE112943 (Fig. 2F). The ORA of the 847 input DEGs demonstrated that interferon signaling (Reactome term: R-HSA-913531) was the most enriched pathway (– log10P = 32.42, Fig. 2G). SLE is characterized by activation of the interferon system, which supports LN as one of the organ manifestations of SLE [32]. Furthermore, using DisGeNET, we found that the LN phenotype was significantly enriched (– log10P = 15, Fig. 2H). Among the enriched diseases, IgA glomerulonephritis is another phenotype associated with kidney disease. Taken together, the 847 DEGs were feasible for exploring disease mechanisms in LN.

### DDGs for LN in tissue and blood samples

**Fig. 3:**
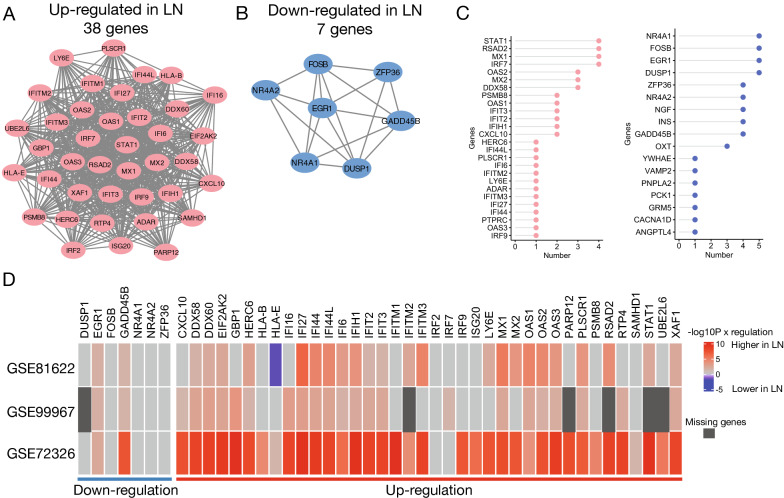
Identification of hub genes for LN. **A** Gene regulatory network constructed for up-regulated DEGs using Cytoscape. **B** Gene regulatory network constructed for down-regulated DEGs using Cytoscape. **C** Dot plot reveals the top 10 regulatory hub genes selected by five CytoHubba methods (selected from up-regulated and down-regulated hub genes). The numbers of times selected by the five algorithms are shown (left and right panels). Five methods: MNC, MCC, EPC, DMNC, and Degree. **D** Heatmap of Wilcoxon’s rank sum test P values derived from the 45 hub genes between LN and control in peripheral blood samples. Color bar indicates –log10-transformed P values that times the sign of regulation. For the blood samples, if the expression is higher in LN, the transformed P value times one; if it is lower in LN, the transformed P value times negative one. The heatmap is separated into up-regulated hub genes (right panel) and down-regulated hub genes (left panel). Insignificant results are colored in light gray. Missing genes are colored in dark gray

To determine a set of genes specific to LN, the top networks of the up-regulated and down-regulated DEGs were identified using the MCODE algorithm. A total of 38 up-regulated and seven down-regulated genes were identified, which together were termed the DDGs for LN (Fig. 3A–B). The five algorithms in CytoHubba were used for DDGs to select the top 10 regulatory genes. We found that *STAT1*, *RSAD2*, *MX1*, and *IRF7* were selected most of the time (n = 4) in the up-regulated gene set, whereas *NR4A1*, *FOSB*, *EGR1*, and *DUSP1* were selected by all five algorithms in the down-regulated gene set (Fig. 3C). Next, blood samples were collected, and the expression levels of DDGs were compared between LN and healthy controls. In addition to some missing genes in GSE99967, the up-regulated DDGs in the tissue samples showed similar differential patterns in the blood samples (Fig. 3D). However, the expression levels of *EGR1* and *GADD45B* were not down-regulated in LN. GSE99967 was removed from subsequent analyses because it contained six missing genes.

### DDGs can discriminate LN from other chronic kidney diseases

**Fig. 4:**
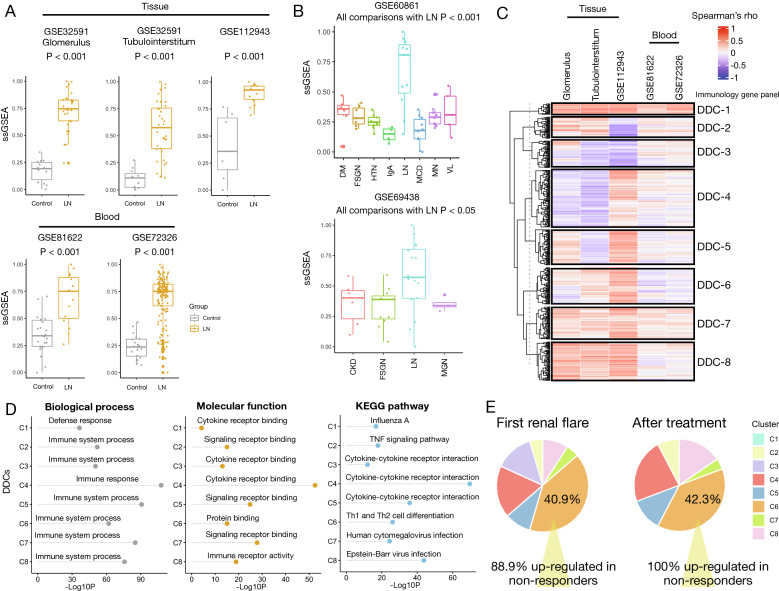
Regulatory-level activity specific for LN. **A** Box plots show differences in ssGSEA scores between LN samples and controls in tissues (GSE32591 and GSE112943) and peripheral blood (GSE81622 and GSE72326). Wilcoxon’s rank sum test P values are shown. **B** Box plots show differences in ssGSEA scores between LN and other chronic renal diseases in GSE60861 (upper panel) and GSE69438 (lower panel). Wilcoxon’s rank sum test P values are shown between LN and other renal diseases. DM: diabetes mellitus. FSGN: focal segmental glomerulonephritis. HTN: hypertension. MCD: minimal change disease. MN: membranous nephropathy. VL: vasculitis. CKD: chronic kidney disease. MGN: membranous glomerulonephritis. **C** Heatmap of correlation coefficients calculated between the ssGSEA scores of hub genes and immunology panel genes in tissue (GSE32591 and GSE112943) and blood samples (GSE81622 and GSE72326). GSE32591 is divided according to tissue origins to evaluate the expression patterns in two tissue compartments. Hierarchical clustering based on the K-means method is performed. Color bar indicates Spearman’s correlation coefficient. Cluster numbers are shown on the right. DDC: disease-defining cluster. **D** Dot plots show enriched GO terms (biological process and molecular function) and KEGG pathways for each eight cluster. –Log10-transformed multiple testing-adjusted P value is shown for each enriched term. **E** Pie plots reveal distribution of DEGs between responders and non-responders in GSE200306 according to the DDCs in renal biopsies obtained at first RF (left panel) and after treatment (right panel). DDC with the largest percentage is annotated. The percentages of genes up-regulated in non-responders are shown in this DDC

The ssGSEA score of the DDGs was calculated to represent the regulation-level activity of LN in each sample. We found striking differences in ssGSEA scores between LN and controls in both tissue and blood samples, with significantly higher ssGSEA scores in LN (all P < 0.001, Fig. 4A). We then performed a permutation test to evaluate whether these differences were specific to DDGs. After repeated selection of random gene sets 1000 times, we found that the difference in the mean values of ssGSEA scores was not statistically significant for a large number of randomly chosen gene sets across the four datasets (Additional file 2: Figure S2). This result indicated that the DDGs could distinguish between the LN and control groups. Next, we found that the ssGSEA scores of the DDGs could also discriminate LN from other chronic kidney diseases, such as diabetic nephropathy, focal segmental glomerulonephritis, membranous glomerulonephritis, and vasculitis, with significantly higher ssGSEA scores in LN. Therefore, this set of DDGs is considered specific for LN.

### Eight DDCs were identified in the immunology gene panel

K-means clustering was performed, and optimal clusters were determined based on the elbow plot. To minimize the total within the sum of squares (WSS), the second elbow was chosen as the optimal cut-off. Eight DDCs were used in our study, and the total WSS was < 100 (Additional file 2: Figure S3 and Additional file 1: Table S2). A hierarchical heatmap partitioned by the eight DDCs is shown in Fig. 4C. Except for DDC-1 and -7, the other DDCs showed various correlation patterns with the ssGSEA of DDGs. Additionally, in tissue samples, most of the immunology panel genes showed moderate to high correlations, suggesting that the expression of these genes might be affected to some degree by the regulation-level activity of the DDGs derived from tissues. GO and KEGG pathway analyses revealed similar top-enriched terms (Fig. 4D). Most of them are related to the immune system process, cytokine receptor binding, and cytokine-cytokine receptor interaction. Of note, the top enriched pathways related to possible pathogenesis in LN were the TNF signaling pathway and Th1 and Th2 differentiation for DDC-2 and -6, respectively [33, 34]. DEGs for treatment response were identified from biopsies obtained at RF and after treatment. We found that approximately 40% of DEGs with |logFC|≥ 1 belonged to DDC-6 (40.9% at RF, 42.3% after treatment, Fig. 4E). Furthermore, 88.9% of DEGs in DDC-6 were up-regulated in non-responders, which increased to 100% after treatment. These findings suggest that subsets of genes in DDC-6 might be potential candidates for predicting treatment response in LN after RF.

### LCK was the top regulatory gene for treatment response in LN after the first RF

**Fig. 5:**
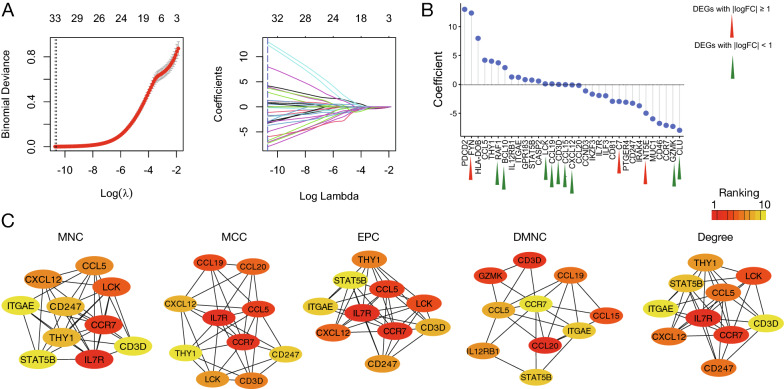
Searching for top-regulatory genes for determining the responders in sub-networks. **A** Binomial deviance plot with lambda as the tuning parameter for DDC-6 (left panel). The red dots are the values of binomial deviance. The gray lines represent the standard error (SE). The vertical dashed lines are the optimal values by the minimum criteria and 1-SE criteria. LASSO coefficient profile of the 33 selected genes in DDC-6 (right panel). **B** Horizontal dot plot demonstrates the coefficients of the 33 selected genes. Red triangle indicates DEGs with |logFC ≥ 1| between responders and non-responders after treatment for LN with the first RF in GSE200306. Green triangle indicates DEGs with |logFC < 1|. **C** Top 10 regulatory gene nodes selected by MNC, MCC, EPC, DMNC, and Degree. Color bar indicates the ranking among the ten selected genes

We then used LASSO to select genes in DDC-6 with predictive value. The parameters were tuned, and genes with non-zero coefficients were identified (Fig. 5A). A total of 33 genes out of 63 DDC-6 genes were selected (Fig. 5B). Among the selected genes, *FYN*, *RAF1*, *BCL10*, *LCK*, *CCL19*, *CD3D*, *CCL15*, *CXCL12*, *C7*, *NT5E*, *GZMK*, and *CLU* were DEGs associated with treatment response in GSE200306. We then applied the five CytoHubba methods to examine the top 10 regulatory nodes in each biological subnetwork. *CCR7* was selected by all algorithms and ranked highly, except for the DMNC method (Fig. 5C). *IL7R* was selected by four algorithms and ranked top in each of them. However, neither gene was differentially expressed between the responders and non-responders. In the next level of searching, *LCK* was found to be one of the DEGs and had higher ranks in MNC, MCC, EPC, and degree. Therefore, in our study, it was considered a key gene in regulating treatment response.

### Estimation of immune cell infiltration according to the expression of LCK reveals distinct clustering results

**Fig. 6:**
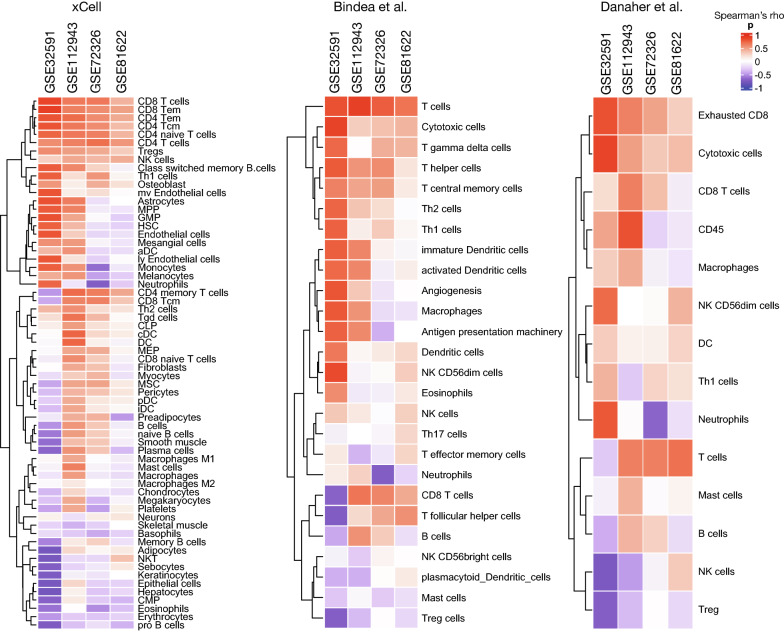
Estimated immune cell infiltrations according to *LCK* expression. Heatmap of Spearman’s correlation coefficients between *LCK* expression and infiltrations of immune cells estimated by three studies (xCell, Bindea et al., and Danaher et al.) in tissue (GSE32591 and GSE112943) and blood samples (GSE72326 and GSE81622). Color bar indicates Spearman’s correlation coefficient. Hierarchical clustering with the euclidean method is performed

ssGSEA scores of gene signatures specific for immune cell infiltration were calculated and correlated with the expression level of *LCK* in both tissue and blood samples with LN. We found moderate to strong positive correlations with most types of T cells, and they were clustered together in hierarchical clustering (Fig. 6). However, *LCK* expression was negatively correlated with Tregs in two of the three studies. Based on the data derived from patients with LN, these findings suggest that *LCK* might modulate immune cell infiltration, especially T cells, in LN.

### LASSO model based on the DDC 6 had the best predictive performance

**Fig. 7:**
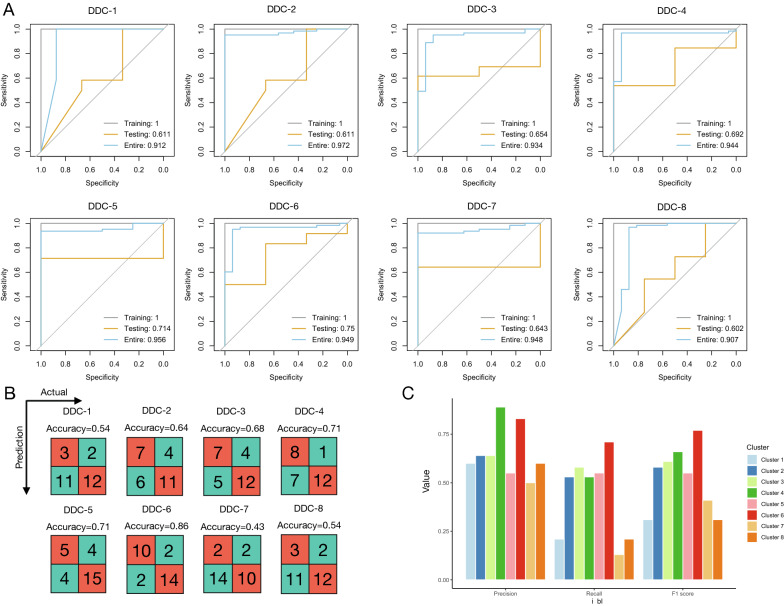
Prediction models of treatment response in the eight DDCs. **A** ROC curves of LASSO prediction models in the training, testing, and entire datasets for the eight DDCs. In each model, predictive genes are selected through LASSO algorithms separately. AUC is shown in each plot. **B** Confusion matrix for the eight prediction models in the validation cohort (GSE113342). **C** Values of precision, recall, and F1 score for the eight prediction models in the validation cohort

Feature selection was performed via LASSO in the eight DDCs to identify predictive genes in each cluster, followed by model training. We found that model performance was the best for DDC-6 (LASSO-DDC-6), with an AUC of 1 (95% CI 1–1), 0.75 (95% CI 0.44–1), and 0.949 (95% CI 0.8836–1) for the training, testing, and entire sets, respectively (Fig. 7A, Additional file 1: Table S3). GSE113342 was used for further validation, and the LASSO-DDC-6 model still outperformed the others, with an accuracy of 0.86 and generally high values of precision (0.83), recall (0.71), and F1 score (0.77) (Fig. 7B–C, Additional file 1: Table S4). These findings indicate that incorporating knowledge specific to a disease into the prediction model is feasible.

## Discussion

We designed a pilot study to evaluate the feasibility of using subsets of nCounter immunology genes to predict treatment response in LN after the first RF. The subsets were defined according to the clustering results, linking the regulatory activity of the hub genes to the immunology gene panel. Overall, through extensive bioinformatics analyses, we identified 45 hub genes that potentially discriminated LN samples from healthy controls and other chronic renal diseases. After considering the systemic nature of SLE, the ssGSEA scores of DDGs calculated in both tissue and blood samples divided the immunology gene panel into eight clusters. With this biologically pre-processed information, a subset of immunology genes was found to be of higher predictive importance and was incorporated into model training. The model performance was high and was validated in an independent dataset. This study demonstrated that our machine learning (ML) model was interpretable and could potentially be used in the clinical setting if more validation data were used.

The initial analysis involved two tissue compartments (glomerulus and tubulointerstitium) in GSE32591. Although they had different DEGs profiles, the two compartments shared a large number of genes involved in interferon signaling. The interferon signaling, especially the type 1 interferon pathway, was well established in the pathogenesis of SLE and LN [35, 36]. This shared pathogenesis was confirmed by Parikh et al., who identified this common dysregulation between the two compartments using renal biopsy from patients with LN [12]. The top hub genes selected via the five Cytohubba algorithms were also supported by current evidence for LN. For example, *STAT1* is involved in the JAK/STAT signaling pathway in response to interferons [37]. Its activation up-regulates *IFI16*, triggering a positive feedback loop that promotes *APOL1*’s expression [38]. *APOL1* overexpression is toxic to podocytes and increases the risk of ESKD in LN. In addition, it has been shown that the protein levels of another interferon-inducible gene, *MX1,* are significantly higher in both the peripheral blood and renal tissues of patients with LN before immunosuppressive treatment, confirming our findings [39]. Down-regulated genes, including *EGR1* and *DUSP1*, were also supported by independent human renal biopsies in LN [20]. Additionally, a permutation test for the ssGSEA scores of the DDGs demonstrated their significance compared with randomly chosen gene sets. Taken together, the DDGs identified in this study represent a valid gene signature.

Based on the 527 immunology genes, evaluation of enriched biological and molecular functions could be redundant, as this panel covers specific genes to address general immune-related gene families, such as the major cytokines, chemokines, and their receptors. Moreover, it does not include all the possible genes for a phenotype to be characterized. For example, infiltration of exhausted CD8 T cells, which is related to prolonged remission in SLE after treatment, was defined by the expression of *LAG3*, *CD244*, and *EOMES* [40, 41]. However, this panel includes only *EOMES*, making it difficult to define the presence of this T cell subgroup. Using our approach, however, several enriched top pathways were identified; and according to literature, the enriched pathways for DDC-2 and DDC-6, TNF signaling pathway, and Th1 and Th2 cell differentiation were most relevant to LN [33, 34]. The benefit of biological knowledge was successfully translated into model performance using LASSO-DDC-6. This indicates that important features (i.e., genes) for determining responders were captured.

ML algorithms are excellent for making successful predictions based on learning the input/output data. In many cases, predictions are accurate even though there is no prior knowledge that directly reflects the underlying physical interactions [42]. However, the interpretation of the models is trivial if the model becomes complex. This is especially true for models such as neural networks, in which interpreting the hidden nodes is challenging, and each node corresponds to a complex nonlinear function of the input data [43]. Furthermore, it is difficult to select the optimal gene sets responsible for classification purposes for bioinformatics analyses that often include DNA microarray datasets. This is due to the small sample size compared to a large number of genes. By accounting for irrelevant and noisy genes, the risk of overfitting increases, which may reduce the generalization of the prediction model [44, 45]. To avoid overfitting, Xiong et al. used several microarray datasets and divided the initial gene pool into clusters based on their structure, followed by LASSO and binary particle swarm optimization [46]. Using this double-filter approach, they could select optimal gene subsets with higher interpretability. For multi-omics data, Xu et al. found LASSO outperformed support vector machine and random forest algorithms [47]. To provide a cost-effective approach for breast cancer detection and patient stratification, they modified the ‘dfmax’ parameter of the *glmnet* function, limiting the maximum number of features in the LASSO model. Instead of incorporating DNA methylation profile and copy number data, they also found using transcriptomic data alone leads to sparse and accurate signatures. In our study, as there were only approximately 500 genes in the immunology panel to determine the responders, we adopted a different approach by projecting the clustering results within the microarray discovery datasets onto the immunology gene panel. The extent of co-regulation of a gene set was quantified by calculating the ssGSEA score. We then linked the LN-specific regulatory activity with the immunology gene expression in the same individual by Spearman’s correlation analysis to estimate the connection their connection. This estimation was indirect, as we used information from microarray data on the nCounter platform. This inherent limitation might be resolved when more features or complete transcriptomic profiles are available. In this way, we could apply novel mix-LASSO model to predict drug response by identifying a smaller number of tissue-specific features, while maintaining the model interpretability and stability for various purposes [48]. Nevertheless, our study suggests that the approach of incorporating knowledge from one platform into another is feasible. However, we have to ensure that platforms being evaluated are sufficiently comparable. Despite the poor correlation between lowly and highly expressed genes in microarray and control-gene-normalized nCounter measurements, the relative expression levels were preserved for most genes [49]. Therefore, we believe that the cross-platform estimation is feasible. Moreover, clustering with the K-means method was applied to the optimal group immunology genes, generating separate gene sets that could be further filtered, reducing the final dimensionality for model construction. In this study, each DDC comprised a maximum of 98 genes (DDC-4) and a minimum of 19 genes (DDC-1), improving the original variable-to-sample ratio. This approach could also be used in samples with complete transcriptomic information to derive interpretable gene sets. Feature scores can then be obtained from gene sets by building simple linear models or feeding them into neural networks. This model transparency could help explain the link between genotypes and phenotypes or assist in discovering novel biomarkers [50, 51]. In the present study, the gene set selected in DDC-6 by LASSO could be a potential gene signature and predictive biomarker.

We also identified that *LCK* was differentially expressed and able to regulate other top-ranked genes in the sub-networks. *LCK* is an Src kinase lymphocyte-specific protein tyrosine kinase that phosphorylates the immunoreceptor tyrosine-based activation motif of CD3ζ after T-cell receptor, which in turn recruits ZAP-70 and causes calcium influx in T cells [52]. In addition to its vital roles in the development, function, and differentiation of T cells, *LCK* is involved in many cellular diseases, such as cell cycle control, proliferation, and differentiation [53]. The activation of T cells, including CD8 T cells, CD4 T follicular helper cells, and subsets of Th17 cells, has been recognized as a key contributor to the pathogenesis of SLE and LN [54]. Ko et al. examined kidney tissues in LN and found increased immunohistochemical staining for CD4 + , CD8 + , and CD68 + in the renal periglomerular area [21]. In our study, *LCK* was up-regulated in non-responders and indirectly correlated with the infiltration of various T cell subtypes. Based on its therapeutic significance in various inflammatory diseases, we hypothesized that *LCK* could be a therapeutic target for LN at RF.

Our study had several limitations. First, the estimation of DDCs was based on different platforms, which could reduce the generalizability of the clustering results, as some genes might not have the same expression pattern or regulatory structure detected by different techniques, which should be carefully addressed by comparing the DDG profiles on the microarray and nCounter platforms. Second, K-means clustering is sensitive to initial conditions. Even though we have evaluated the clustering results for many times to identify the optimal clustering results, there was still slight difference for each random start. However, one of the advantages of K-means clustering is its efficiency and the ability of handling larger datasets. In the future work, bootstrap sampling may be helpful to improve its problem. Third, only one ML algorithm was used in this pilot study. Other models suitable for transparency and interpretability will be of interest in the future. Nevertheless, the performance of our model was comparable to that of a recent ML-based prediction model [15]. In their study, they trained a variety of ML algorithms with 246 subjects to develop prediction models for 1-year proteinuria and estimated glomerular filtration rate (eGFR) in LN. They found the combined model with traditional clinical data and novel urine biomarkers for eGFR had the best performance in training and validation datasets and the AUC was near 0.7, which was slightly lower than our model. Fourth, the validation cohort had a small sample size, and replicates of patients were present due to inclusion of different tissue compartments. Finally, we did not investigate the gene expression for cell junctions as they are important factors related to proteinuria, which is a clinical determination of RF [55]. Further experiment and the inclusion of larger validation samples are required.

Treatment of LN has been faced with many challenges. One of the challenges is the poor target tissue distribution for immunosuppressive drugs [56]. However, with the emergence of nanotechnology, potential nanomaterials with special physiochemical properties are being developed and applied for treating various diseases including glomerulonephritis [57]. With better penetration of loaded drugs, clinical outcomes could be more likely associated with their therapeutic effects in the absence of tissue barrier, which may lead to better prediction model performance due to stronger connection between drugs and outcomes.

## Conclusion

In conclusion, we applied integrated bioinformatics analyses and incorporated knowledge of LN-specific regulatory activity into the training of ML models. The model performance was acceptable, and interpretability increased. In addition, we identified *LCK* to be of vital importance in determining LN responders after RF. This therapeutic target needs to be experimentally verified in future studies.

## Supplementary Information

**Additional file 1: Table S1.** Details of datasets used. **Table S2.** Gene sets for each DDC. **Table S3.** Model performance based on each DDC. **Table S4.** Prediction performance validated in GSE113342.

**Additional file 2: Figure S1.** Diagram of study design. **Figure S2.** Permutation test to confirm the validity of the hub genes in LN. Scatter plots show ssGSEA scores calculated from 1000 random selection of 45 genes. Each data point in the plot indicates data for a randomly generated gene set. The red point highlighted is calculated from the hub gene in our study. x-axis is absolute mean difference =| mean(ssGSEA-LN)—mean(ssGSEAcontrol)| and y-axis is P-value estimated by Wilcoxon’s rank sum test of ssGSEA score between LN and control (green lines represent P = 0.05). **Figure S3.** Elbow plot of K-means clustering. Horizontal lines indicate the first and second elbow.

## Data Availability

All data used could be obtained from public sources (details in Additional file 1: Table S1). Transcriptomic profiles of LN were downloaded from the GEO database (https://www.ncbi.nlm.nih.gov/geo/) under accession numbers GSE32591 and GSE112943. Blood samples from patients with LN were obtained from the GEO database under accession numbers GSE81622, GSE99967 and GSE72326. Datasets that contained transcriptomic data of LN and other chronic renal diseases were retrieved from the GEO database under accession numbers GSE60861 and GSE69438. Datasets for establishment of prediction models and validation were downloaded from the GEO database under accession numbers GSE200306 and GSE113342.

## Abbreviations

LN: Lupus nephritis
RF: Renal flare
ML: Machine learning
DDGs: Disease-defining genes
ssGSEA: Single-sample gene set enrichment analysis
LASSO: Least absolute shrinkage and selection operator
ROC: Receiver operating characteristic
AUC: Area under curve
CI: Confidence interval
DDCs: Disease-defining clusters
SLE: Systemic lupus erythematosus
CYC: Cyclophosphamide
MMF: Mycophenolate mofetil
ESKD: End stage kidney disease
RMA: Robust multi-array average
PCA: Principal component analysis
DEG: Differentially expressed genes
BH: Benjamini-Hochberg
ORA: Over-representation analysis
GO: Gene Ontology
PPI: Protein–Protein interaction
STRING: Search tool for the retrieval of interacting genes
MCODE: Molecular complex detection
MNC: Maximum neighborhood component
MCC: Maximal clique centrality
EPC: Edge percolated component
DMNC: Density of maximum neighborhood component
KEGG: Kyoto Encyclopedia of Genes and Genomes
SMOTE: Synthetic minority oversampling technique
WSS: Within the sum of squares
FC: Fold change
