# Supplementary material for: Incorporating knowledge of disease-defining hub genes and regulatory network into a machine learning-based model for predicting treatment response in lupus nephritis after the first renal flare
Source: J Transl Med. 2023 Feb 3;21:76. doi: 10.1186/s12967-023-03931-z (PMC9898995; doi:10.1186/s12967-023-03931-z)
Supplement: Supplementary file 1 — Additional file 1: Table S1. Details of datasets used. Table S2. Gene sets for each DDC. Table S3. Model performance based on each DDC. Table S4. Prediction performance validated in GSE113342. [file 12967_2023_3931_MOESM1_ESM.pdf]

Table S1. Details of datasets used.

| Study | Array name | Platform | N | Patient | Control | Tissue Type | Purpose |
|---|---|---|---|---|---|---|---|
| GSE32591 | Affy_HGU133A_CDF_ENTREZG_10 | GPL14663 | 93 | 64 | 29 | Renal biopsy | Discovery |
| GSE112943 | Illumina HumanHT-12 V4.0 expression beadchip | GPL10558 | 21 | 14 | 7 | Renal biopsy | Discovery |
| GSE81622 | Illumina HumanHT-12 V4.0 expression beadchip | GPL10558 | 40 | 15 | 25 | Blood | Validation |
| GSE99967 | Affymetrix Human Gene 2.0 ST Array | GPL21970 | 46 | 29 | 17 | Blood | Validation |
| GSE72326 | Illumina HumanHT-12 V4.0 expression beadchip | GPL10558 | 178 | 157 | 21 | Blood | Validation |
| GSE60861 | Agilent-026652 Whole Human Genome Microarray 4x44K v2 | GPL13497 | 65 | 11 | 54 (Other kidney diseases) | Renal biopsy | Validation |
| GSE69438 | Affymetrix Human Genome U133 Plus 2.0 Array | GPL11670 | 37 | 16 | 21 (Other kidney diseases) | Renal biopsy | Validation |
| GSE200306 | nCounter NanoString Human Immunology v2 | GPL21847 | 68 | 58 | 10 | Renal biopsy | Model construction |
| GSE113342 | nCounter NanoString Human Immunology v2 | GPL21847 | 44 | 28 | 16 | Renal biopsy | Validation of model |
| Total | | | 592 | 392 | 200 | | |

Table S2. Gene sets for each DDC.

| | |
|---|---|
| DDC-1 | MX1, STAT1, IFIT2, IFI16, GBP1, SERPING1, TNFSF10, CXCL10, IFI35, IFITM1, IFIH1, TAP1, IRF7, C1QB, HLA-B, PSMB8, CD163, CCL8, MYD88 |
| DDC-2 | HLA-C, CASP3, HLA-DQB1, C1R, VCAM1, BLNK, ITGAM, MSR1, LILRA5, TNFSF15, IRF1, FCGR2B, CXCL1, CASP8, TNFRSF1B, ATG12, S1PR1, C1QBP, TLR8, SLAMF7, ICAM1, HLA-A, TLR1, PYCARD, BST2, LAMP3, CD24, CD55, BCAP31, CD59, EDNRB, CHUK, HFE, KLRK1, ATM, UBE2L3, HRAS |
| DDC-3 | CCRL2, PML, LILRB4, CD83, BTK, NLRP3, PDGFB, TNFSF12, CEACAM8, RELB, CLEC4E, PTPN22, IL1RAP, LILRB5, RAG1, CXCL2, TCF7, IFNB1, IL22, EGR1, LTBR, SRC, TNFSF8, CEACAM6, FOXP3, BCL2, GFI1, TOLLIP, MASP1, CD1A, TGFBR1, LILRA4, TAL1, FCAR, KIR3DL3, STAT5A, ZBTB16, TNFRSF8, IKZF2, CUL9, PLA2G2E, NCAM1, IRF3, SMAD3, IL11RA, CD160 |
| DDC-4 | STAT2, NFIL3, CISH, CTSG, IRF4, BCL3, RELA, KLRC4, MAPKAPK2, MIF, MME, SLC2A1, ITGB1, CD22, CXCR3, NFKBIA, BCL2L11, XCL1, NFATC3, IL6R, TNFSF11, IL1RL2, CD8B, XCR1, IL26, AIRE, TRAF3, IL5, CDKN1A, CCL22, TNFRSF10C, CIITA, CD70, SOCS3, ARG1, CASP10, LTB4R, CRADD, TGFB1, NFKB2, MAPK11, MAP4K1, ICAM4, IL21, ICAM5, RORC, IL23A, IL2RA, CSF1, CD3E, B3GAT1, CD6, CAMP, BST1, IL3, JAK3, LILRB3, LTA, CCL16, C8B, POU2F2, C8A, CSF2, TRAF4, VTN, C6, C4BPA, C8G, NFATC1, ITGA2B, IL13, ICOSLG, PAX5, MASP2, ARG2, DEFB1, IFNG, CD28, TNFRSF9, IL12B, RAG2, PRDM1, LIF, IL18RAP, IL2, NOS2, IL1A, DPP4, CD82, NOD1, FKBP5, IFNA2, CR1, TNFSF4, KIR3DL1, PLA2G2A, IL4, CD40LG |
| DDC-5 | IL1RN, IL18R1, IL2RG, CMKLR1, BATF3, PSMB5, IRF5, CD209, PDGFRB, ICOS, IL4R, CCL13, CD4, ETS1, SLAMF1, CCR6, CARD9, IKZF1, CXCR6, LILRA2, PLAUR, IL1B, LILRB1, TRAF6, CFP, CD80, TNF, ITGAX, IL21R, STAT6, TYK2, ITGA5, PRKCD, TRAF2, LILRA1, NOTCH1, PTAFR, MAP4K2, CCL18, IKBKE, BATF, SELE, IL1RL1, TNFRSF4, IRAK3, DUSP4, PSMB7, CD8A, CCR10, CD19, CD79B, CCL4, MARCO, ITGAL, IL16, TBX21, ZAP70, SELPLG, GNLY, IL2RB, TRAF1 |
| DDC-6 | IL13RA1, CD81, SPP1, IL32, CXCL12, C3, CFH, HLA-DQA1, MALT1, CD3D, NT5E, CCL5, CCND3, BCL10, IRAK4, CCL20, THY1, GPR183, TP53, CD9, CTSC, ICAM3, GZMK, LCK, MAPK1, PDCD2, IGF2R, PRF1, TNFRSF14, STAT4, IL7R, MBP, CFI, NOTCH2, TRAF5, IKZF3, CCL11, CCL15, CLU, SMAD5, CD247, KIT, KLRF1, CCL19, RAF1, PIGR, C7, ILF3, HLA-DOB, GPI, MUC1, CD46, CASP2, C5, CCR7, MAF, STAT5B, ITGAE, SIGIRR, IL12RB1, FYN, PTGER4, ABCB1 |
| DDC-7 | CCL2, CEACAM1, S100A9, LTF, TLR4, SELL, FAS, MR1, NOD2, TAP2, C2, CLEC4A, ITGA4, NCF4, SYK, BCL6, CD58, CXCL11, PTPN6, LILRB2, TICAM1, CCR2, TAPBP, IL18, IL1R2, C1S, XBP1, TLR7, JAK2, S100A8, TNFRSF17, STAT3, IL15, MAPK14, PTK2, CXCR4, KCNJ2, IRAK1, ITGA6, NFKB1, ABL1, CXCL9, IL1R1, PTGS2, FCGRT, TNFAIP3, CX3CL1, TLR5, CD164, PTPN2, ATG7, CEBPB, PLAU, |

| | |
|---|---|
| | SOCS1, MCL1, CTNNB1 |
| DDC-8 | C1QA, CASP1, HLA-DRB1, PSMB9, CD14, FCER1G, TLR2, CD53, TBK1, ZEB1, TCF4, LCP2, PSMC2, CFD, ITGB2, HLA-DRA, B2M, CTSS, ARHGDIB, PECAM1, HLA-DMB, HLA-DPA1, CFB, IL6ST, IL7, PSMB10, TLR3, CD74, ATG5, HLA-DMA, CSF1R, ICAM2, TGFBI, CD48, IL10RA, HLA-DPB1, MAP4K4, CX3CR1, FADD, LITAF, GZMB, TGFBR2, ENTPD1, BID, CD86, LAIR1, IFNAR2, AHR, CLEC7A, CD40, CD34, FN1, IFNAR1, CDH5, APP, IRF8, CD44, IDO1, ADA, PSMD7, CD36, BAX, PPARG, CCR5, GATA3, IKBKB, GZMA |

DDC: Disease-defining cluster

Table S3. Model performance based on each DDC.

| DDC | Training set AUC (95% CI) | Testing set AUC (95% CI) | Entire set AUC (95% CI) |
|---|---|---|---|
| DDC-1 | 1.0 (1.00-1.00) | 0.61 (0.39-1.00) | 0.91 (0.82-1.00) |
| DDC-2 | 1.0 (1.00-1.00) | 0.61 (0.10-0.81) | 0.97 (0.86-1.00) |
| DDC-3 | 1.0 (1.00-1.00) | 0.65 (0.10-0.83) | 0.93 (0.84-1.00) |
| DDC-4 | 1.0 (1.00-1.00) | 0.69 (0.48-1.00) | 0.94 (0.84-1.00) |
| DDC-5 | 1.0 (1.00-1.00) | 0.71 (0.10-0.89) | 0.96 (0.85-1.00) |
| DDC-6 | 1.0 (1.00-1.00) | 0.75 (0.44-1.00) | 0.95 (0.88-1.00) |
| DDC-7 | 1.0 (1.00-1.00) | 0.64 (0.10-0.82) | 0.95 (0.88-1.00) |
| DDC-8 | 1.0 (1.00-1.00) | 0.60 (0.10-0.79) | 0.90 (0.81-1.00)) |

DDC: Disease-defining cluster

Table S4. Prediction performance validated in GSE113342.

| DDC | Precision | Recall | F1 score |
|---|---|---|---|
| DDC-1 | 0.6 | 0.21 | 0.31 |
| DDC-2 | 0.64 | 0.53 | 0.58 |
| DDC-3 | 0.64 | 0.58 | 0.61 |
| DDC-4 | 0.89 | 0.53 | 0.66 |
| DDC-5 | 0.55 | 0.55 | 0.55 |
| DDC-6 | 0.83 | 0.71 | 0.77 |
| DDC-7 | 0.5 | 0.13 | 0.41 |
| DDC-8 | 0.6 | 0.21 | 0.31 |

DDC: Disease-defining cluster
