# Supplementary material for: Incorporating knowledge of disease-defining hub genes and regulatory network into a machine learning-based model for predicting treatment response in lupus nephritis after the first renal flare
Source: J Transl Med. 2023 Feb 3;21:76. doi: 10.1186/s12967-023-03931-z (PMC9898995; doi:10.1186/s12967-023-03931-z)
Supplement: Supplementary file 2 — Additional file 2: Figure S1. Diagram of study design. Figure S2. Permutation test to confirm the validity of the hub genes in LN. Scatter plots show ssGSEA scores calculated from 1000 random selection of 45 genes. Each data point in the plot indicates data for a randomly generated gene set. The red point highlighted is calculated from the hub gene in our study. x-axis is absolute mean difference =| mean(ssGSEA-LN)—mean(ssGSEAcontrol)| and y-axis is P-value estimated by Wilcoxon’s rank sum test of ssGSEA score between LN and control (green lines represent P = 0.05). Figure S3. Elbow plot of K-means clustering. Horizontal lines indicate the first and second elbow. [file 12967_2023_3931_MOESM2_ESM.pdf]

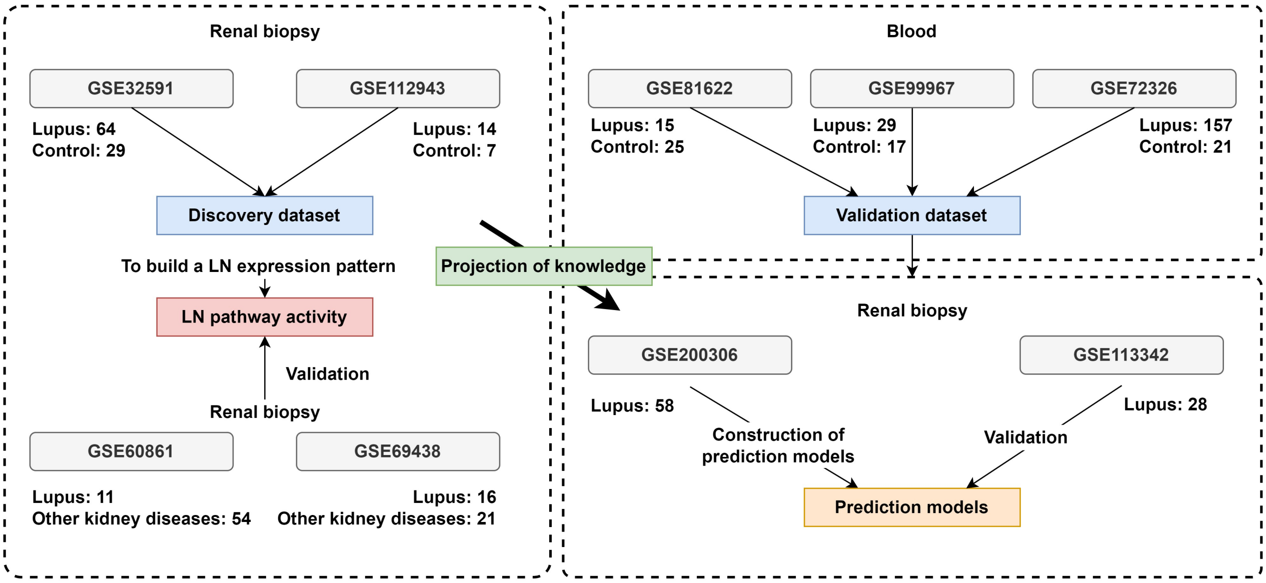

Figure S1. Diagram of study design.

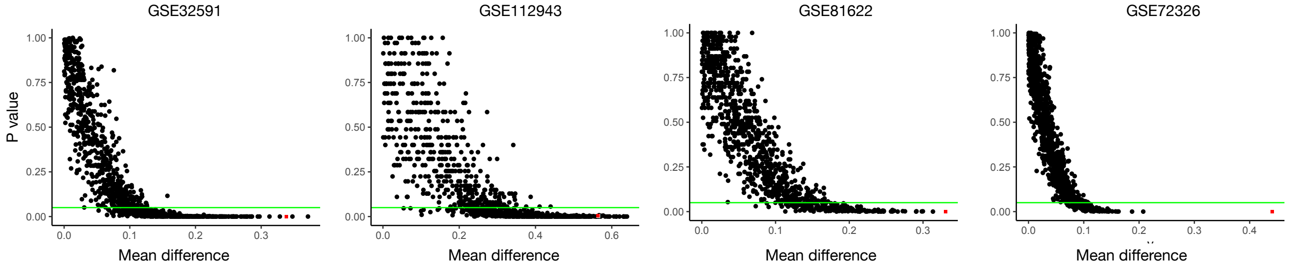

Figure S2. Permutation test to confirm the validity of the hub genes in LN. Scatter plots show ssGSEA scores calculated from 1000 random selection of 45 genes. Each data point in the plot indicates data for a randomly generated gene set. The red point highlighted is calculated from the hub gene in our study. x-axis is absolute mean difference = | mean(ssGSEA-LN) - mean(ssGSEA-control)| and y-axis is P-value estimated by Wilcoxon's rank sum test of ssGSEA score between LN and control (green lines represent P-value = 0.05).

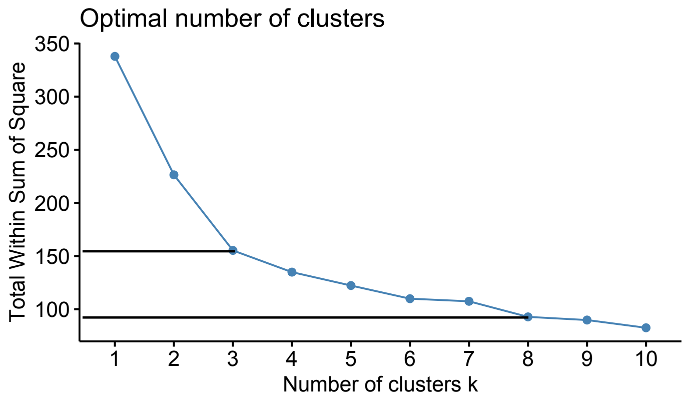

Figure S3. Elbow plot of K-means clustering. Horizontal lines indicate the first and second elbow.
